# Neuroprotective Effects of Necrostatin-1 Against Oxidative Stress–Induced Cell Damage: an Involvement of Cathepsin D Inhibition

**DOI:** 10.1007/s12640-020-00164-6

**Published:** 2020-01-21

**Authors:** Danuta Jantas, Jakub Chwastek, Beata Grygier, Władysław Lasoń

**Affiliations:** 1 Department of Experimental Neuroendocrinology, Maj Institute of Pharmacology Polish Academy of Sciences, Smętna Street 12, 31-343 Kraków, Poland; 2 Present Address: Department of Neurochemistry, Maj Institute of Pharmacology Polish Academy of Sciences, Smętna Street 12, 31-343 Kraków, Poland; 3 Present Address: Department of Immunology, Faculty of Biochemistry, Biophysics and Biotechnology, Jagiellonian University, Gronostajowa 7 Street, 30-387 Kraków, Poland

**Keywords:** Hydrogen peroxide, SH-SY5Y cells, HT-22 cells, Caspase-3, Glutamate, Pepstatin A

## Abstract

Necroptosis, a recently discovered form of non-apoptotic programmed cell death, can be implicated in many pathological conditions including neuronal cell death. Moreover, an inhibition of this process by necrostatin-1 (Nec-1) has been shown to be neuroprotective in in vitro and in vivo models of cerebral ischemia. However, the involvement of this type of cell death in oxidative stress–induced neuronal cell damage is less recognized. Therefore, we tested the effects of Nec-1, an inhibitor of necroptosis, in the model of hydrogen peroxide (H_2_O_2_)-induced cell damage in human neuroblastoma SH-SY5Y and murine hippocampal HT-22 cell lines. The data showed that Nec-1 (10–40 μM) attenuated the cell death induced by H_2_O_2_ in undifferentiated (UN-) and neuronal differentiated (RA-) SH-SY5Y cells with a higher efficacy in the former cell type. Moreover, Nec-1 partially reduced cell damage induced by 6-hydroxydopamine in UN- and RA-SH-SY5Y cells. The protective effect of Nec-1 was of similar magnitude as the effect of a caspase-3 inhibitor in both cell phenotypes and this effect were not potentiated after combined treatment. Furthermore, the non-specific apoptosis and necroptosis inhibitor curcumin augmented the beneficial effect of Nec-1 against H_2_O_2_-evoked cell damage albeit only in RA-SH-SY5Y cells. Next, it was found that the mechanisms of neuroprotective effect of Nec-1 against H_2_O_2_-induced cell damage in SH-SY5Y cells involved the inhibition of lysosomal protease, cathepsin D, but not caspase-3 or calpain activities. In HT-22 cells, Nec-1 was protective in two models of oxidative stress (H_2_O_2_ and glutamate) and that effect was blocked by a caspase inhibitor. Our data showed neuroprotective effects of the necroptosis inhibitor, Nec-1, against oxidative stress–induced cell damage and pointed to involvement of cathepsin D inhibition in the mechanism of its action. Moreover, a cell type–specific interplay between necroptosis and apoptosis has been demonstrated.

## Introduction

Necroptosis, a form of non-apoptotic programmed cell death, was shown to be activated in various models of acute (stroke, traumatic brain, or spinal cord injuries) and chronic (Alzheimer’s disease (AD), Parkinson’s disease (PD), and amyotrophic lateral sclerosis (ALS)) neurodegenerative conditions (Caccamo et al. 2017; Re et al. 2014; Vieira et al. 2014; Yin et al. 2015). This cell death pathway can be distinguished from apoptosis based on morphological and biochemical criteria. The cell dying by necroptosis is characterized by the loss of plasma membrane integrity, swelling of cellular organelles, and lack of typical nuclear fragmentation. Moreover, it can be activated by members of the tumor necrosis factor (TNF) family (TNFR1, TNFR2, TRAILR1, TRAILR2), Fas ligand, toll-like receptors (TLRs), lipopolysaccharides (LPS), pathogens (bacteria and viruses), genotoxic stress, glutamate (Glu), or calcium overload, and is thought to be a caspase-independent process. A crucial step in initiation and execution of necroptosis involves the activation of receptor-interacting serine/threonine-protein (RIP) kinases (RIP1 and RIP3) and formation of the necrosome complex (Degterev et al. 2014; Zhao et al. 2015).

In recent years, various inhibitors of necroptosis have been experimentally tested in relation to various human pathologies and used for mechanistic investigation of this process (Degterev et al. 2005, 2008, 2013; Delehouzé et al. 2017; Do et al. 2017; Li et al., 2018; Xie et al. 2013). Among them, necrostatin-1 (Nec-1; *methyl-thiohydantoin-tryptophan*) was the first to be identified and since then used the most frequently (Degterev et al. 2005, 2008). The neuroprotective potential of Nec-1 and its analogs in cellular and animal models of ischemia is rather well supported (Chavez-Valdez et al. 2012, 2016; Degterev et al. 2005; Li et al. 2018; Ni et al. 2018; Northington et al. 2011; Xu et al. 2010a; Yang et al. 2017a; Yin et al. 2015; Zhan et al. 2019; Zhang et al. 2016). Moreover, in an in vitro setting, Nec-1 attenuated the neuronal cell damage induced by various harmful agents, e.g., oaubain, glutamate (Glu), N-methyl-D-aspartate (NMDA), 6-hydroxydopamine (6-OHDA), rotenone, 1-methyl-4-phenylpyridinium ion (MPP+), methamphetamine, aluminum (Al), 24(S)-hydroxycholesterol, cholesterol, staurosporine, and doxorubicin (Funakoshi et al. 2016; Ito et al. 2017; Jantas et al. 2014a; Li et al. 2011; Wang et al. 2014; Wu et al. 2015; Xiong et al. 2016; Xu et al. 2007; Yamanaka et al. 2011; Zhang et al. 2013). It should be noted that the protective effect of Nec-1 in the above studies was only partial, suggesting the participation of other than necroptosis cell death programs which can vary depending on the type of neuronal injury as has already been shown by in vivo (ischemia, traumatic brain injury, spinal cord injury, retina injury) and in vitro studies (e.g., Al-, iron-, 6-OHDA-, or β-amyloid-induced neurotoxicity) (Askalan et al. 2015; Chinskey et al. 2014; Dai et al. 2013; Dong et al. 2012; Liu et al. 2014; Rosenbaum et al. 2010; Qinli et al. 2013; Wu et al. 2015; Xu et al. 2010a; Zhang et al. 2008). Consequently, a potential synergistic effect has been proposed after combined treatment with various cell death–specific inhibitors (Koshinuma et al. 2014; Xu et al. 2010a).

With the aim to extend a neuroprotective portfolio of Nec-1, in the present study, we tested the hypothesis that this compound protects human neuroblastoma SH-SY5Y cells against the hydrogen peroxide (H_2_O_2_)- and 6-hydroxydopamine (6-OHDA)-induced cell damage. The used experimental models are widely accepted as cellular models of PD, where elevated oxidative stress is a significant contributor to neuropathology of this disease (Agholme et al. 2010; Castelli et al. 2019; Cenini et al. 2019; Cheung et al. 2009; Miloso et al. 2004). Based on our previous experiences, where neuronal differentiation of SH-SY5Y cells could mask or limit a protective effect of the tested agents (Jantas et al. 2014b, 2015b), we hypothesized that Nec-1 will afford a greater protection to undifferentiated (UN-SH-SY5Y) cells in comparison with retinoic acid (RA)-differentiated (RA-SH-SY5Y) ones. Although necrosis and/or apoptosis have been shown to participate in the H_2_O_2_-induced cell damage in SH-SY5Y cells (Chwastek et al. 2017; Jantas et al. 2015a), the involvement of necroptosis has not been studied in detail, yet. In order to test a potential interplay between apoptosis and necroptosis in our models, we co-treated cells with the caspase-3 inhibitor, Ac-DEVD-CHO, and Nec-1. Since previous studies showed neuroprotective effects of curcumin in various types of neuronal cell damage (Mhillaj et al. 2019; Szczepanowicz et al. 2016) and the involvement of both apoptosis and necroptosis inhibition has been suggested to be associated with the curcumin-mediated protection (Dai et al. 2013; Wang et al. 2017; Xu et al. 2019), we studied a potential synergism in neuroprotective effects of Nec-1 and curcumin (Curc) in UN- and RA-SH-SY5Y cells. Next, we searched for putative mechanisms involved in neuroprotection mediated by Nec-1 by measuring the apoptotic markers (caspase-3 activity, AIF translocation), calpain activity (145 kDa cleavage product of spectrin α II) and lysosomal permeability (cathepsin D activity). For confirmation of the results in another type of neuronal cells, we tested the effect of Nec-1 and its possible synergism with a caspase inhibitor (Z-VAD-fmk) in the mouse hippocampal HT-22 cells exposed to H_2_O_2_ or glutamate (Glu).

## Materials and Methods

### Drugs and Reagents

Dulbecco’s Modified Eagle’s Medium (DMEM), FluoroBrite™ DMEM, and fetal bovine serum (FBS) were from Gibco (Invitrogen, Paisley, UK). The Cytotoxicity Detection Kit and BM Chemiluminescence Western Blotting Kit were from Roche Diagnostic (Mannheim, Germany). Caspase-3 (Ac-DEVD-AMC) and cathepsin D (MOCA-Gly-Lys-Pro-Ile-Leu-Phe-Phe-Arg-Leu-Lys(Dnp)-D-Arg-NH2) fluorogenic substrates were obtained from Enzo Life Sciences (NY, USA). Caspase-1 substrate (Ac-YVAD-AMC) and inhibitor (Ac-YVAD-CHO) were obtained from Promega (Madison, WI, USA). Primary antibodies, anti-spectrin α II (sc-48382), anti-AIF (sc-5586), anti-cathepsin D (sc-6486), anti-GAPDH (sc-25778), and MW standards (sc-2035), and secondary antibodies (sc-2004, sc-2005, sc-2020, and sc-2030) were purchased from Santa Cruz Biotechnology Inc. (CA, USA). All other reagents were from Sigma (Sigma-Aldrich Chemie GmbH, Germany).

### SH-SY5Y Cell Culture

The human SH-SY5Y neuroblastoma cells (ATCC, passages 5–20) were grown in DMEM supplemented with 10% (v/v) heat-inactivated FBS and 100 units/ml of penicillin and 100 μg/ml of streptomycin as described previously (Jantas et al. 2015b). Cells were maintained at 37 °C in a saturated humidity atmosphere containing 95% air and 5% CO_2_. After reaching an 80% confluence, cells were counted with a LUNA™ Automatic Cell Counter (Logos Biosystems, Inc., Korea) and seeded at a density of 5 × 10^4^, 2.5 × 10^5^, and 1 × 10^6^ cells per well into 96-, 24-, and 6-well plates, respectively. To obtain differentiated cells (RA-SH-SY5Y), the cells were plated at a half of the densities mentioned above and cultured in medium supplemented with retinoic acid (RA, 10 μM) for 6 days, during which the culture medium was changed every 2 days. One day before treatment, the culture medium for undifferentiated (UN-SH-SY5Y) and RA-SH-SY5Y cells was replaced with DMEM containing antibiotics and 1% (v/v) FBS.

### HT-22 Cell Culture

The immortalized mouse hippocampal cell line HT-22 (passages 302–308; kind gift from Prof. Carsten Culmsee, Institute of Pharmacology and Clinical Pharmacy, University of Marburg, Germany) was grown in DMEM supplemented with 10% (v/v) FBS and 1% (v/v) penicillin/streptomycin as reported previously (Chwastek et al. 2017; Dolga et al. 2013). After trypsinization, the cells were counted (LUNA™ Automatic Cell Counter, Logos Biosystems, Inc., Korea) and seeded into 96-well plates at a density of 2 × 10^4^ and 8 × 10^3^ cells/well for H_2_O_2_- and Glu-induced cell damage, respectively. Cells were maintained at 37 °C in a saturated humidity atmosphere containing 95% (v/v) air and 5% (v/v) CO_2_. One day before treatment, the culture medium for the model of H_2_O_2_-evoked cell damage was replaced with DMEM containing 1% (v/v) FBS and antibiotics. For Glu-mediated oxytosis, the medium contained 10% (v/v) FBS (Dolga et al. 2013).

### Cell Treatment

The cells were pre-treated with various concentrations of Nec-1 (1–40 μM) for 30 min followed by 24 h exposure to cell damaging factors (H_2_O_2_, 6-OHDA, Glu). The chosen concentrations of Nec-1 were based on literature search and included not active (< 1 μM) and RIP1-specific (> 3 μM) concentrations (Degterev et al. 2008; Ito et al. 2017; Yamanaka et al. 2011; Yang et al. 2017b, 2019). For comparison of the Nec-1 effects with other protectants, the antioxidant, N-acetylcysteine (NAC, 1 mM), and curcumin (Curc, 5 μM) were used. The effective concentrations of H_2_O_2_ (0.25 and 0.5 mM for UN- and RA-SH-SY5Y cells, respectively; 1 mM for HT-22 cells) were established in our previous studies, in which this agent reduced cell viability by approximately 50% (Chwastek et al. 2017; Jantas et al. 2015a). The chosen concentrations of 6-OHDA (0.1 and 0.2 mM for UN- and RA-SH-SY5Y cells, respectively) or Glu (3 mM) were based on the published literature (Cheung et al. 2009; Dolga et al. 2013; Tieu et al. 1999). For mechanistic studies, inhibitors of calpain (MDL28170; 10 μM)), cathepsin D (pepstatin A; PsA; 0.2 μM), caspase-3 (Ac-DEVD-CHO; 20 μM), caspase-1 (Ac-YVAD-CHO; 20 μM), and pan-caspase inhibitor (Z-VAD-fmk; 20 μM) were used.

Nec-1 (100 mM), MDL28170 (10 mM), PsA (10 mM), Ac-DEVD-CHO (10 mM), Ac-YVAD-CHO (10 mM), Z-VAD-fmk (10 mM), and Curc (10 mM) stock solutions were prepared in DMSO. The final solutions of the tested chemicals were prepared in distilled water except for those of Curc which were prepared in a mixture of dH_2_O and DMSO (1:1). The H_2_O_2_ stock solutions (25 and 50 mM) were prepared freshly from stabilized 30% (w/w) hydrogen peroxide diluted in distilled water. The 6-OHDA stock solutions (10 and 20 mM) were prepared in distilled water immediately before use. All agents were added to the culture medium at the indicated concentrations under light limited conditions. Each experimental set of the control cultures was supplemented with the appropriate vehicles, and the solvent was present in cultures at a final concentration of 0.1% (v/v).

### Cell Viability Assay

Cell viability of UN- and RA-SH-SY5Y or HT-22 cells growing in 96-well plate format after 24 h of particular treatments was quantified using a tetrazolium salt colorimetric assay with 3-[4,5-dimethylthiazol-2-yl]-2,5-diphenyltetrazolium bromide (MTT) as described previously (Jantas et al. 2015a). The data were normalized to the vehicle-treated cells (100%) and expressed as a percent of the control ± SEM established from 3 to 11 independent experiments with 5 replicates.

### LDH Release Assay

The level of lactate dehydrogenase (LDH) released into culture media from UN-, RA-SH-SY5Y, or HT-22 cells after 24 h of treatment with Nec-1 and H_2_O_2_ was measured with Cytotoxicity Detection Kit (Roche) as described previously (Jantas et al. 2015a). The data were normalized to the vehicle-treated cells and expressed as a percent of the control ± SEM from 3 to 11 independent experiments with 5 replicates.

### PI Staining and Flow Cytometry

To confirm the results obtained by biochemical cell viability/toxicity assays, the SH-SY5Y and HT-22 cells were stained with propidium iodide (PI) according to the method described previously (Jantas et al. 2015a). A total of 1 × 10^4^ cells were analyzed using a BD FACS Canto II System and BD FACSDiva™ v5.0.1 Software (BD Biosciences) in the fluorescence channel for PerCP-Cy5-5-A (red fluorescence). The cells exhibiting loss of cell membrane integrity (PI positive) represent necrotic and late apoptotic cells. Data are presented as a percentage of PI-positive cells (± SEM) established from 3 to 5 independent experiments with 2 replicates.

### CalceinAM/Hoechst 33342 Staining and Image Analysis

In order to assess the morphology and viability of the UN- and RA-SH-SY5Y cells after 9 and 18 h of treatment with Nec-1 (20 μM) and H_2_O_2_ (0.25 and 0.5 mM for UN- and RA-SH-SY5Y cells, respectively), we performed live cell imaging by employing a double staining of cells with cell-permeable CalceinAM (marker of viable cells) and Hoechst 33342 (nuclei marker) dyes, as described previously (Fraczek-Szczypta et al. 2018). The cells after labeling were placed in FluoroBrite™ DMEM and were evaluated by using an inverted fluorescence microscope (AxioObserver, Carl Zeiss) with an excitation wavelength of 480 nm (CalceinAM) and 355 nm (Hoechst 33342) equipped with a black-white camera (Axio-CamMRm, Carl Zeiss). Five microphotographs for each panel (480 or 355 nm) were taken for each tested group in duplicates for each endpoint (9 or 18 h) from 2 independent experiments. The numbers of pyknotic (condensed and/or fragmented) and healthy nuclei were counted semi-manually from a 355-nm panel using AxioVison software for each taken microphotograph, averaged from 5 photos per well and the data are presented as the mean ± SEM. To evaluate the impact of the tested agents on neurite morphology, the neurite length was measured from a 480-nm panel using Simple Neurite Tracer (an ImageJ add-on software) as described previously (Fraczek-Szczypta et al. 2018). The lengths of ten random neurites per image, 3 images per well, and 2 wells per experimental group were measured. The data are presented as the mean neurite length ± SEM (in μm) from 2 independent experiments.

### Caspase-3 and Caspase-1 Activity Assays

The caspase-3 activity in UN- or RA-SH-SY5Y cells growing in 6-well format and treated for 9 or 18 h with H_2_O_2_ and Nec-1 was measured using fluorogenic substrate Ac-DEVD-AMC (50 μM) as described in detail previously (Jantas et al. 2015a). Caspase-3 inhibitor, Ac-DEVD-CHO (20 μM), was used to verify the assay specificity. Moreover, we measured caspase-3 and caspase-1 activities in RA-SH-SY5Y cells after 9 and 18 h of treatment with Nec-1 (20 μM), Curc (5 μM), and H_2_O_2_ (0.5 mM). Caspase-1 activity was measured according to a similar procedure as caspase-3 but with a different substrate (Ac-YVAD-AMC, 50 μM) and inhibitor (Ac-YVAD-CHO, 20 μM) as described previously (Jantas et al. 2018a). The data (expressed as mean relative fluorescence units, RFU) first were normalized to protein level (measured by BCA method) and next calculated as a percent of vehicle-treated cells and presented as the mean ± SEM from 2 to 3 separate experiments with 2 repetitions each.

### Cathepsin D Activity Assay

Cathepsin D activity in UN- and RA-SH-SY5Y cells treated for 18 h with H_2_O_2_ and Nec-1 was measured using a fluorometric method employing the fluorogenic substrate AMC-Gly-Lys-Pro-Ile-Leu- Phe-Phe-Arg-Leu-Lys(Dnp)-D-Arg-NH2 as described previously (Chwastek et al. 2017). PsA (0.2 μM) was used as a positive control for the assay. Cathepsin D activity expressed in RFU first was calculated per milligram of protein and next normalized to the vehicle-treated cells; it is shown as the mean ± SEM from 3 to 4 independent experiments with 2 replicates.

### Western Blot

For Western blot analysis of spectrin α II cleavage products in whole cell lysates, UN- or RA-SH-SY5Y cells were cultured in 6-well plates and pre-treated for 30 min with Nec-1 (20 μM) or calpain inhibitor MDL28170 (10 μM) followed by 14 h exposure to H_2_O_2_ (0.25 and 0.5 mM for UN- and RA-SH-SY5Y cells). For cathepsin D protein measurement (43 and 33 kDa) in whole cell lysates, UN- and RA-SH-SY5Y cells were pre-treated with Nec-1 (20 μM) for 30 min followed by 18 h exposure to H_2_O_2_. For apoptosis inducing factor (AIF) measurement in cytosolic fraction, UN-SH-SY5Y cells were pre-treated with Nec-1 (1 and 20 μM) for 30 min followed by 14 h exposure to H_2_O_2_. The whole cell lysates and cytosolic fractions (for measurement of AIF expression) were prepared as previously described (Jantas et al. 2015a). Equal amounts of protein were separated on 7% (w/v) (spectrin α II) or 10% (w/v) (AIF, cathepsin D) SDS polyacrylamide gels and transferred onto PVDF membranes. After blocking with 5% (w/v) nonfat milk in TBST, the membranes were incubated overnight with primary antibodies diluted at 1:500 (AIF, cathepsin D) and 1:1000 (spectrin α II, GAPDH) in 1% (w/v) nonfat milk. The amount of GAPDH was determined on the same membrane on which the protein of interest was measured, by stripping and reprobing the membrane as described previously (Jantas et al. 2015a). Data from 2 to 3 independent experiments were normalized to the protein loading control (GAPDH) and are expressed as fold of control (± SEM).

## Statistical Analysis

Data were analyzed using the Statistica software (StatSoft Inc., Tulsa, OK, USA). The analysis of variance (one-way ANOVA) and post hoc Tukey’s test for multiple comparisons were used to show statistical significance with assumed *P* < 0.05. For comparison of Nec-1 protective efficiency between UN- and RA-SH-SY5Y cells, a mean area under the curve (AUC) was calculated and statistically analyzed with (GraphPad Prism 7.04) with assumed *P* < 0.05. The unpaired *t* test was also used for comparison of basal activities of caspase-3 or cathepsin D in UN- and RA-SH-SY5Y cells.

## Results

### Neuroprotective Effects of Nec-1 Against H_2_O_2_- and 6-OHDA-Induced Cell Damage in UN- and RA-SH-SY5Y Cells: the Impact of Cell Differentiation State

**Fig. 1:**
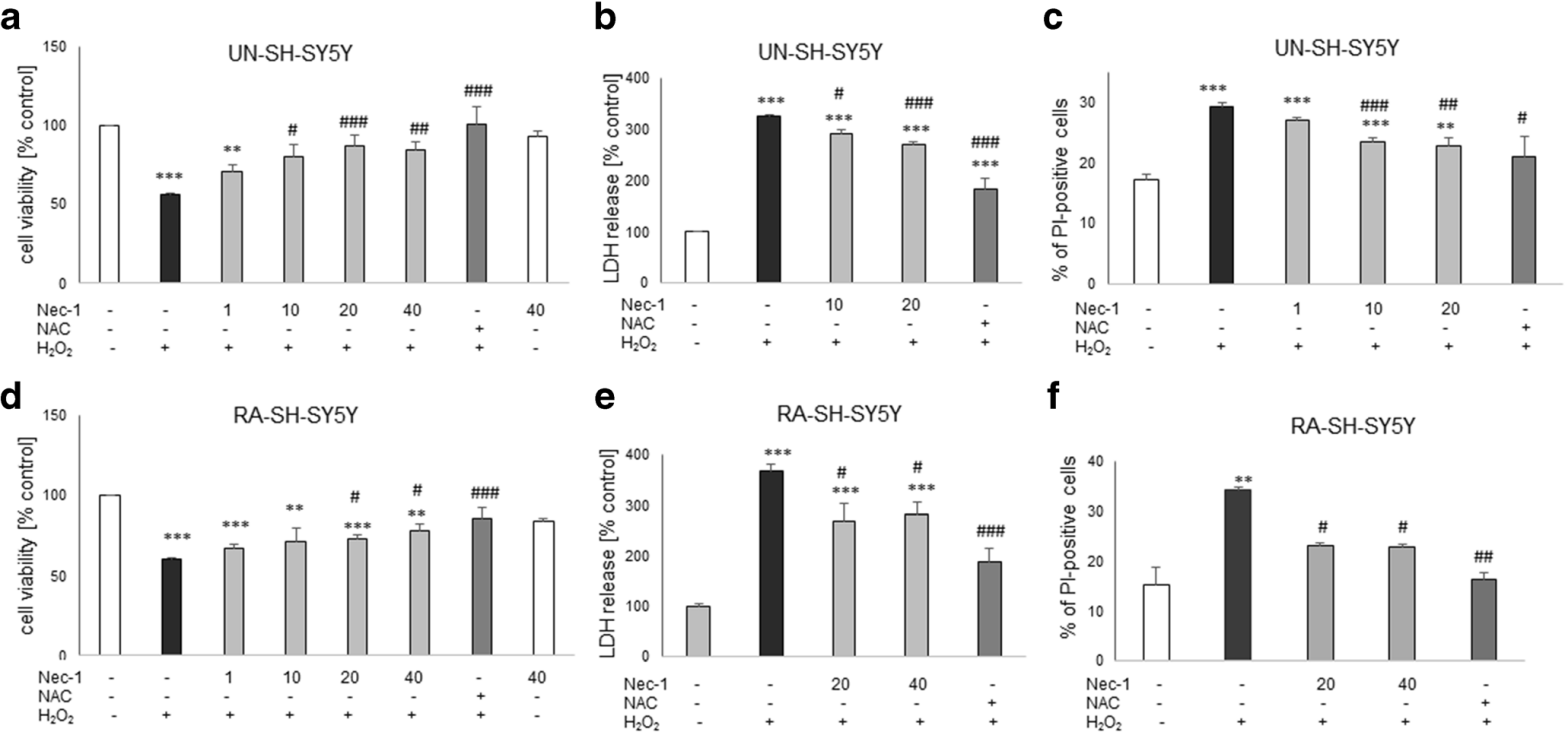
The effect of necrostatin-1 on H_2_O_2_-induced cell damage in UN- and RA-SH-SY5Y cells. UN- and RA-SH-SY5Y cells (**a**–**c** and **d**–**f**, respectively) were pre-treated for 30 min with necrostatin-1 (Nec-1; 1–40 μM) followed by 24 h of treatment with H_2_O_2_ (0.25 and 0.5 mM for UN- and RA-SH-SY5Y, respectively). As a positive control for the assays, we used antioxidant N-acetylcysteine (NAC, 1 mM) which was given concomitantly with the cell damaging factor. **a**, **d** Results of cell viability assessment in UN-(**a**) and RA-(**d**) SH-SY5Y cells measured by the MTT reduction assay. Data were normalized to vehicle-treated cells (control) and are presented as the mean ± SEM from 3 to 11 separate experiments with 5 repetitions each. (**b**, **e**) Results of cell toxicity assessment in UN-(**b**) and RA-(**e**) SH-SY5Y cells measured by the LDH release assay. Data were normalized to vehicle-treated cells (control) and are presented as the mean ± SEM from 4 to 11 separate experiments with 5 repetitions each. **c**, **f** Flow cytometry results of propidium iodide (PI)-stained UN- (**c**) and RA (**f**) SH-SY5Y cells after 24 h of cell treatment. Data are presented as the mean ± SEM of PI-positive cells from 3 to 5 independent experiments with 2 replicates. **P < 0.01 and ***P < 0.001 vs. vehicle-treated cells; ^#^*P* < 0.05, ^##^*P* < 0.01, and ^###^*P* < 0.001 vs. H_2_O_2_-treated cells

**Fig. 2:**
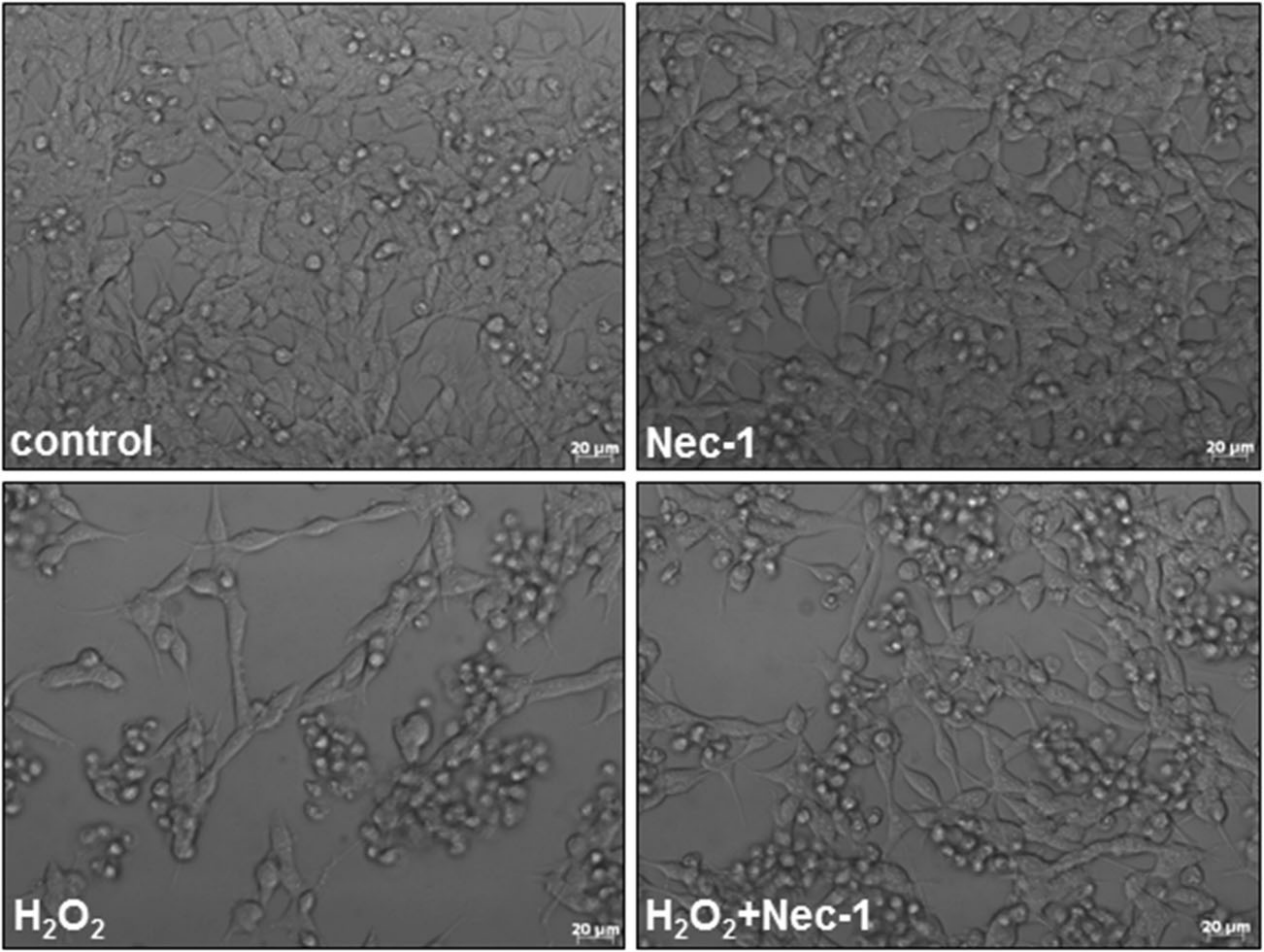
Representative DIC (*differential interference contrast*) images of UN-SH-SY5Y cells treated for 24 h with necrostatin-1 (Nec-1, 20 μM) and hydrogen peroxide (H_2_O_2_, 0.25 mM)

**Fig. 3:**
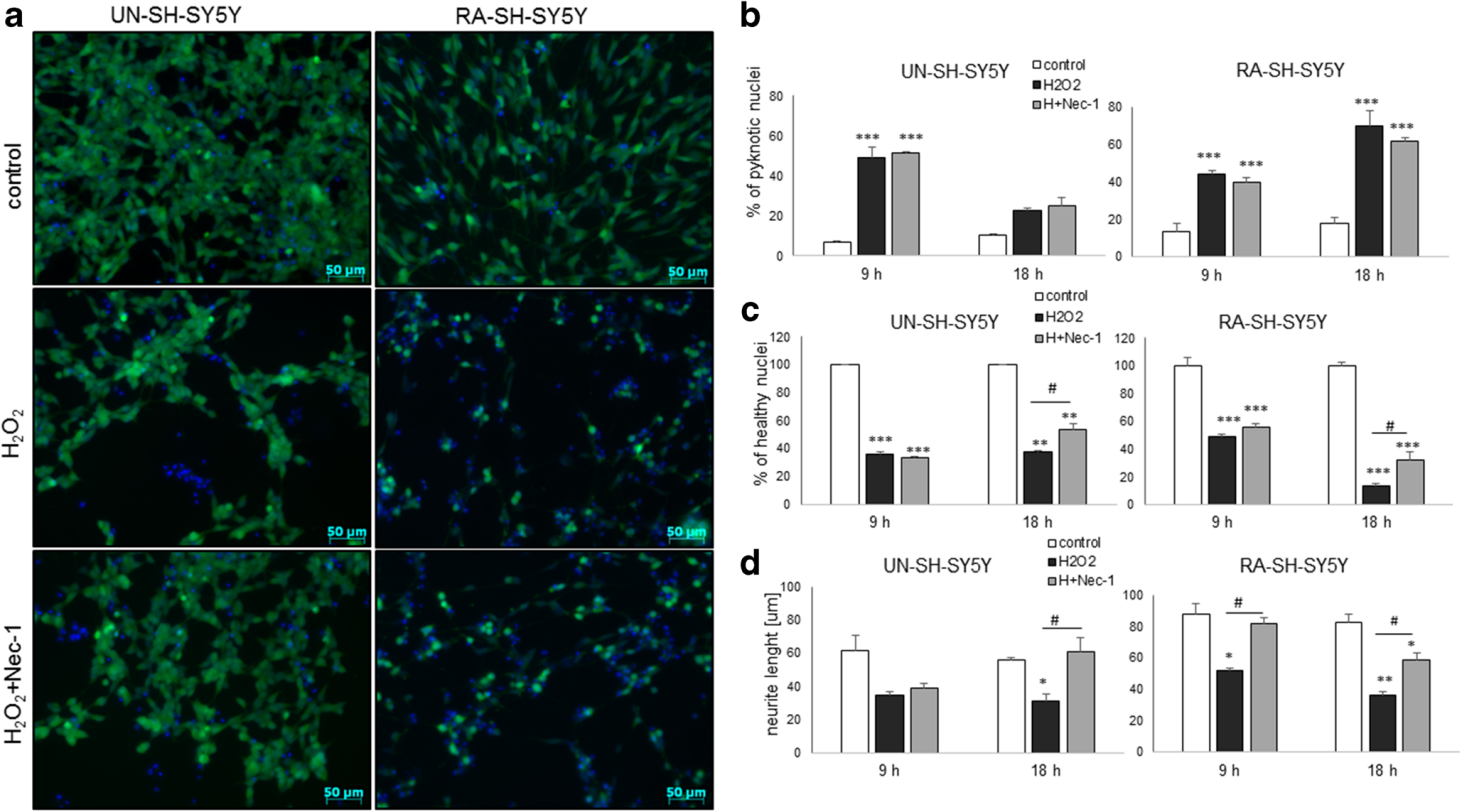
**a** Representative microphotographs of UN- and RA-SH-SY5Y cells double-stained with CalceinAM/Hoechst 33342 after 18 h of treatment with necrostatin-1 (Nec-1; 20 μM) and hydrogen peroxide (H_2_O_2_; 0.25 and 0.5 mM for UN- and RA-SH-SY5Y cells, respectively). **b**, **c** An estimation of number of pyknotic (**b**) and healthy nuclei (**c**) from Hoechst 33342 staining. Nuclei showing bright blue florescence (condensed or fragmented) staining were semi-manually counted and presented as the mean percentage of pyknotic nuclei/all nuclei ± SEM or percentage of healthy nuclei (normalized to control group) from two independent experiments with two replicates. **d** Quantification of neurite length (in μm) from CalceinAM staining using Simple Neurite Tracer (an ImageJ add-on software). The lengths of ten random neurites per image, 3 images per well, and 2 wells per experimental group were measured. The data are presented as a mean neurite length ± SEM (in μm) from 2 independent experiments. Statistical analysis was performed with one-way ANOVA with Tukey’s post hoc test independently for each time point. ***P < 0.05, **P < 0.01, and ***P < 0.001 vs. vehicle-treated cells; ^#^*P* < 0.05 vs. H_2_O_2_-treated cells

Twenty-four hours of treatment with Nec-1 at up to 40 μM was safe for UN- or RA-SH-SY5Y cells as confirmed by cell viability assay (Fig. 1a, d). Nec-1 (10–40 μM) attenuated the cell damage induced by H_2_O_2_ in UN- and RA-SH-SHY5Y cells (Fig. 1a, d) with a significantly higher protection (measured as a mean area under the curve (AUC)) mediated in the former cell phenotype (AUC = 95.26 ± 5.74 and AUC = 44.82 ± 4.34 for UN- and RA-SH-SY5Y, respectively; *t* test, *P* = 0.0009). It should be noticed that the neuroprotection mediated by the antioxidant, N-acetylcysteine (NAC, 1 mM), was comparable with the effect of Nec-1 at its higher concentrations (20 and 40 μM) (Fig. 1). The neuroprotective action of Nec-1 and NAC against H_2_O_2_ was confirmed by the LDH release assay (Fig. 1b, e) and by PI staining in both cell phenotypes (Fig. 1c, f). Moreover, the beneficial effect of Nec-1 on cell viability was also morphologically confirmed by *differential interference contrast* (DIC) images (Fig. 2) and by CalceinAM staining (Fig. 3a). Additionally, we showed a significant increase in the number of pyknotic nuclei after treatment of UN-SH-SY5Y cells (after 9 h) and RA-SH-SY5Y cells (after 9 and 18 h) with H_2_O_2_ which was not changed by Nec-1 (20 μM) pre-treatment at any of the tested time points (Fig. 3b). However, we observed that Nec-1 partially protected the cells against H_2_O_2_-induced reduction in the number of healthy nuclei which was observed after 18 h but not after 9 h of treatment in both cell phenotypes (Fig. 3c). Next, we measured the impact of Nec-1 pre-treatment on H_2_O_2_-evoked neurite shortening after 9 and 18 h of treatment. In UN-SH-SY5Y cells, we found a significant reduction in this parameter after 18 h of treatment with H_2_O_2_ which was completely blocked by Nec-1 pre-treatment (Fig. 3d, left panel). In the case of RA-SH-SY5Y cells, the H_2_O_2_ evoked reduction in neurite length after 9 and 18 h of treatment which was significantly reduced by Nec-1 (Fig. 3d, right panel).

To confirm the neuroprotective potential of Nec-1 in SH-SY5Y cells, we also used another oxidative stress–based model employing 6-OHDA. We observed a partial protection mediated by Nec-1 in UN-SH-SY5Y (20 and 40 μM) and RA-SH-SY5Y (40 μM) cells (Table 1) against 6-OHDA-induced cell damage with a slight tendency towards a higher protection in the former cell type (AUC = 24.64 ± 4.15 and AUC = 14.11 ± 2.91 for UN- and RA-SH-SY5Y, respectively; *t* test, *P* = 0.0968).

**Table 1:** The effect of necrostatin-1 on 6-OHDA-induced cell damage in UN- and RA-SH-SY5Y cells

	UN-SH-SY5Y	RA-SH-SY5Y
Control	99.9 ± 0.1	100.0 ± 0.0
6-OHDA	62.8 ± 0.6***	68.6 ± 0.2***
+ Nec-1 20	72.0 ± 2.6***^,#^	70.2 ± 2.0***
+ Nec-1 40	78.2 ± 5.6***^, ##^	81.2 ± 3.8**^, #^
+ NAC	81.4 ± 4.7***^, ###^	83.2 ± 4.0**^, #^

UN- and RA-SH-SY5Y cells were pre-treated for 30 min with necrostatin-1 (Nec-1; 20 and 40 μM) followed by 24 h of treatment with 6-hydroxydopamine (6-OHDA, 0.1 and 0.2 mM for UN- and RA-SH-SY5Y cells, respectively)

An antioxidant N-acetylcysteine (NAC, 1 mM) was given to cells concomitantly with 6-OHDA. The MTT reduction assay was employed for cell viability assessment

Data were normalized to the vehicle-treated cells and are presented as the mean ± SEM from 4 separate experiments with 5 repetitions each

**P < 0.01 and ***P < 0.001 vs. vehicle-treated cells

^#^*P* < 0.05, ^##^*P* < 0.01, and ^##^*P* < 0.001 vs. 6-OHDA-treated cells

### Neuroprotective Effects of Nec-1 Against Oxidative Stress–Induced Cell Damage in HT-22 Cells

**Fig. 4:**
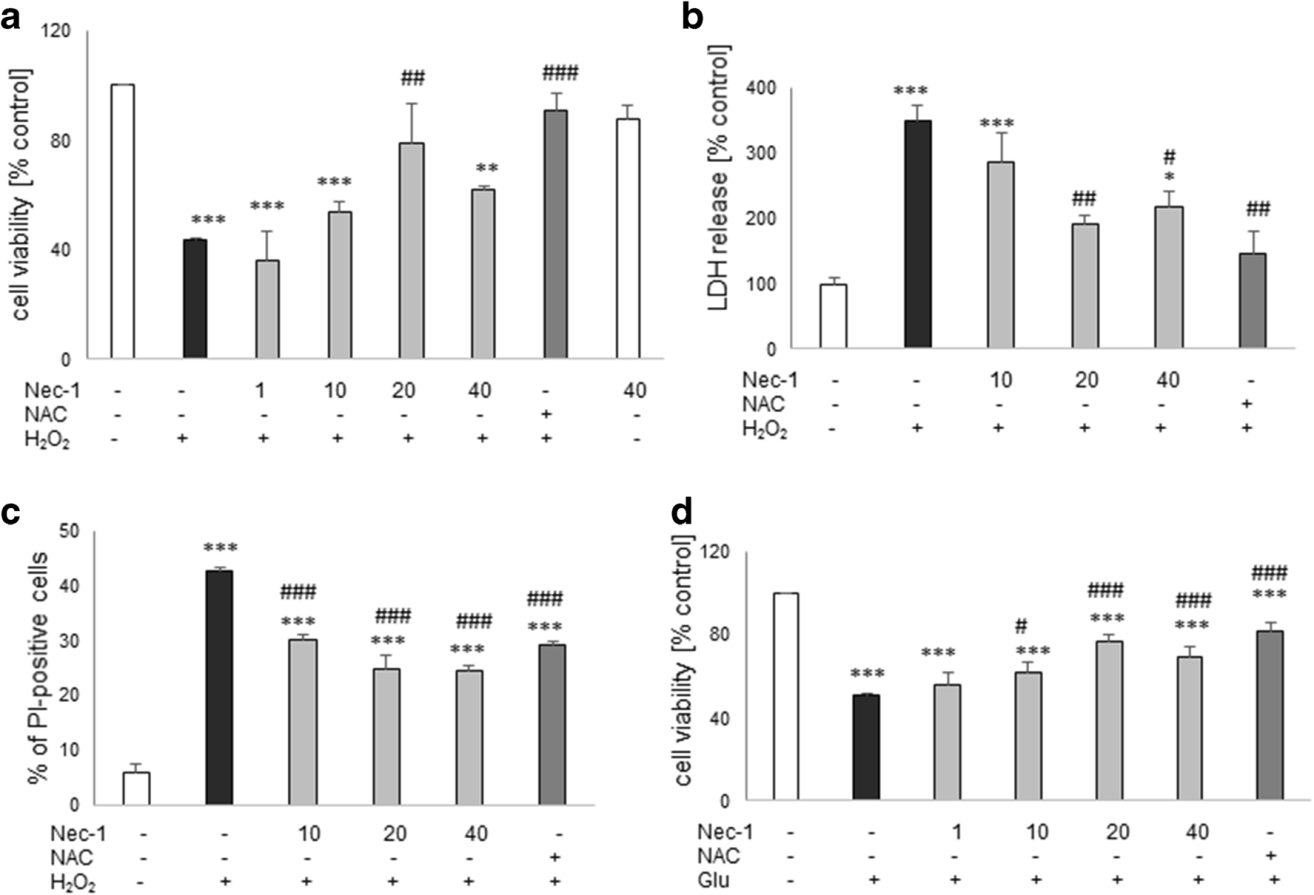
The effect of necrostatin-1 on H_2_O_2_- (**a**–**c**) or glutamate- (Glu, **d**) induced cell damage in hippocampal HT-22 cells. The cells were pre-treated for 30 min with necrostatin-1 (Nec-1; 1–40 μM) followed by 24 h of treatment with H_2_O_2_ (1 mM) or Glu (3 mM). As a positive control for the assays, we used antioxidant N-acetylcysteine (NAC, 1 mM) which was given concomitantly with the cell damaging factors. **a**, **d** Results of cell viability assessment in the model of cell damage induced by H_2_O_2_- (**a**) and Glu (**d**) measured by the MTT reduction assay. Data were normalized to vehicle-treated cells (control) and are presented as the mean ± SEM from 3 to 10 separate experiments with 5 repetitions each. **b** Results of cell toxicity assessment in HT-22 cell exposed to H_2_O_2_ and Nec-1 measured by the LDH release assay. Data were normalized to vehicle-treated cells (control) and are presented as the mean ± SEM from 3 to 5 separate experiments with 5 repetitions each. **c** Flow cytometry results of propidium iodide (PI)-stained HT-22 cells after 24 h of cell treatment with H_2_O_2_ and Nec-1. Data are presented as the mean ± SEM of PI-positive cells from 3 to 4 independent experiments with 2 replicates. *P < 0.05, **P < 0.01, and ***P < 0.001 vs. vehicle-treated cells; ^#^*P* < 0.05, ^##^*P* < 0.01, and ^###^*P* < 0.001 vs. H_2_O_2_- or Glu-treated cells

Twenty-four hours of treatment with Nec-1 at up to 40 μM was safe for HT-22 cells (Fig. 4a). Nec-1 attenuated the HT-22 cell damage induced by H_2_O_2_ at concentrations of 20 μM, 20–40 μM, and 10–40 μM in the MTT reduction, LDH release, and PI staining assays, respectively (Fig. 4a–c). Moreover, Nec-1 significantly attenuated the Glu-evoked cell damage in the concentration range 10–40 μM and that effect was similar to NAC-mediated protection (Fig. 4d).

### The Effect of Combined Treatment with Nec-1 and Caspase-3 Inhibitor in UN- and RA-SH-SY5Y Cells

**Fig. 5:**
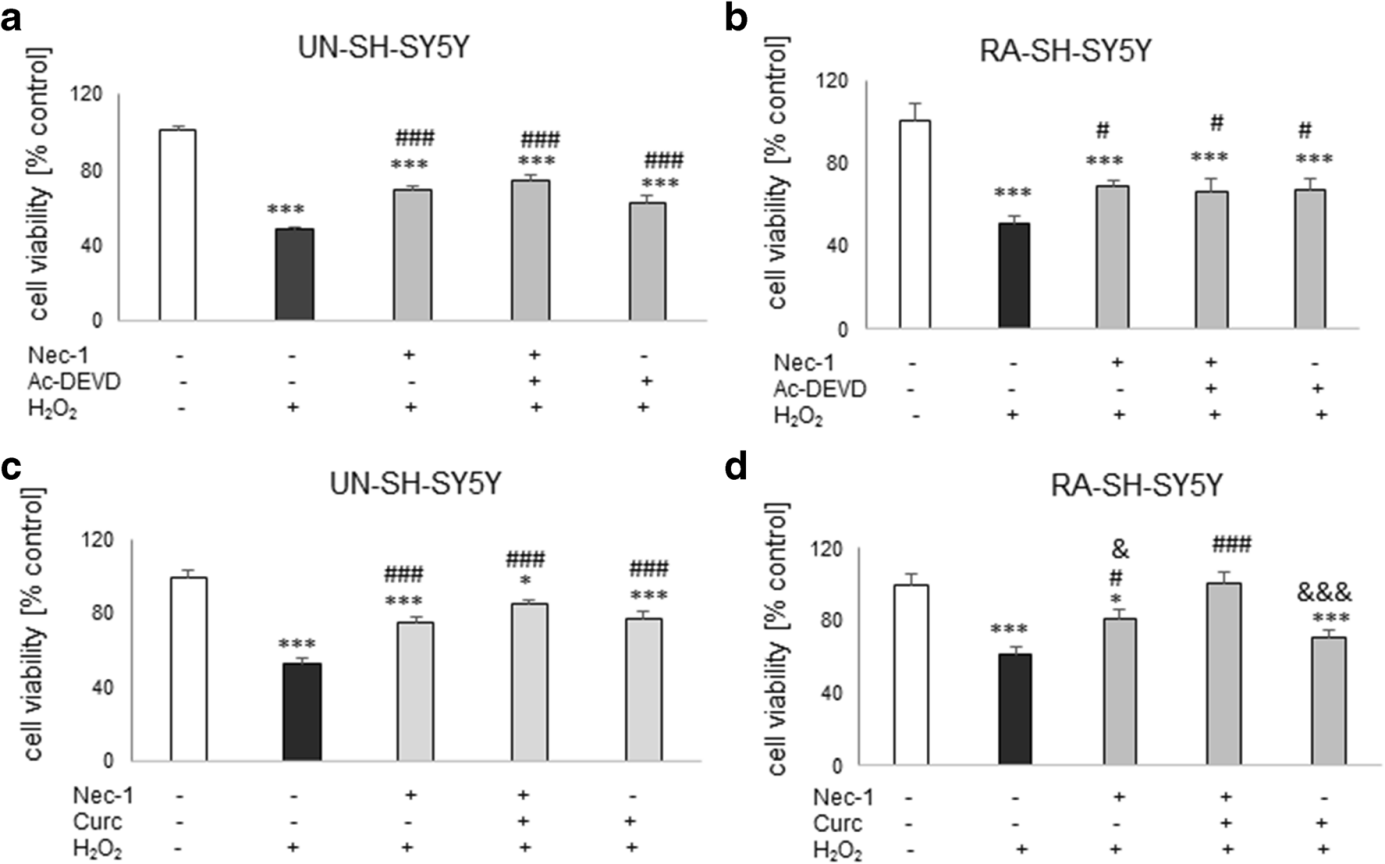
The effect of combined treatment with necrostatin-1 (Nec-1) and caspase-3 inhibitor (Ac-DEVD-CHO) (**a**, **b**) or Nec-1 and curcumin (Curc) against the hydrogen peroxide-induced cell damage in UN- (**a**, **c**) and RA- (**b**, **d**) SH-SY5Y cells. The cells were pre-treated for 30 min with Nec-1 (20 μM) and Ac-DEVD-CHO (20 μM) or Nec-1 (20 μM) and Curc (5 μM) alone or in combination followed by 24 h of treatment with H_2_O_2_ (0.25 mM and 0.5 mM for UN- and RA-SH-SY5Y cells). Cell viability was estimated by the MTT reduction assay, and data were normalized to vehicle-treated cells (control) and are presented as the mean ± SEM from 3 separate experiments with 5 repetitions each. *P < 0.05 and ***P < 0.001 vs. vehicle-treated cells; ^#^*P* < 0.05 and ^###^*P* < 0.001 vs. H_2_O_2_-treated cells; ^&^*P* < 0.05 and ^&&&^*P* < 0.001 vs. H_2_O_2_ + Nec-1 + Curc-treated cells

**Fig. 6:**
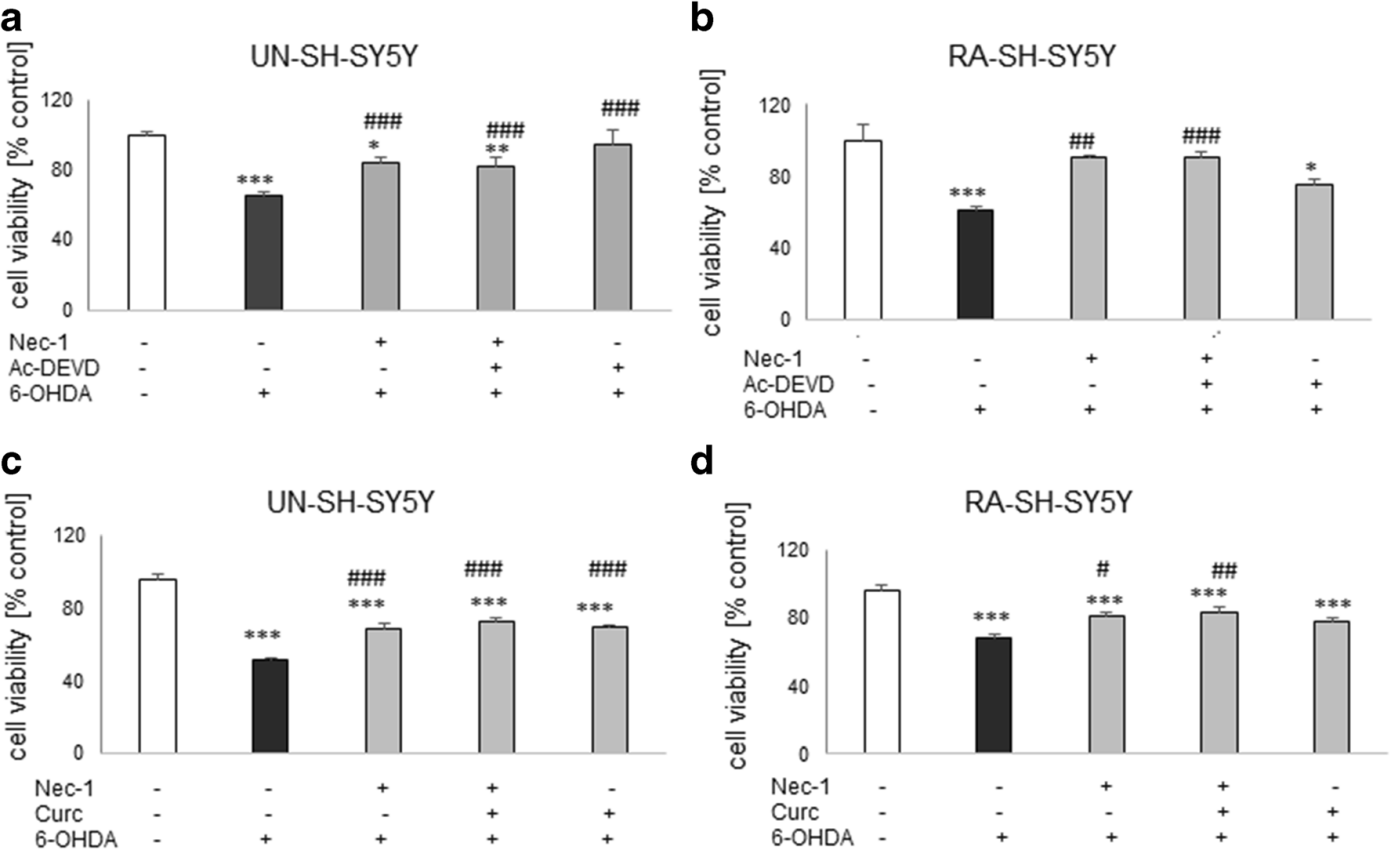
The effect of combined treatment with necrostatin-1 (Nec-1) and caspase-3 inhibitor (Ac-DEVD-CHO) (**a**, **b**) or Nec-1 and curcumin (Curc) (**c**, **d**) against the 6-OHDA-induced cell damage in UN- (**a**, **c**) and RA- (**b**, **d**) SH-SY5Y cells. The cells were pre-treated for 30 min with Nec-1 (20 μM) and Ac-DEVD-CHO (20 μM) or Nec-1 (20 μM) and Curc (5 μM) alone or in combination followed by 24 h of treatment with 6-OHDA (0.1 and 0.2 mM for UN- and RA-SH-SY5Y cells, respectively). Cell viability was estimated by the MTT reduction assay, and data were normalized to vehicle-treated cells (control) and are presented as the mean ± SEM from 3 separate experiments with 5 repetitions each. *P < 0.05, **P < 0.01, and ***P < 0.001 vs. vehicle-treated cells; ^#^*P* < 0.05, ^##^*P* < 0.01, and ^###^*P* < 0.001 vs. H_2_O_2_-treated cells

Since in our study we used the cell damage models connected with induction of apoptosis, and on the other hand we observed protective effects of necroptosis inhibitor Nec-1, we have speculated that combination of a caspase-3 inhibitor with Nec-1 could increase the extent of protection when compared with their effects when given separately. In UN- and RA-SH-SY5Y cells, we observed a similar range of protection mediated by Nec-1 and Ac-DEVD-CHO against H_2_O_2_-evoked cell damage when drugs were administrated separately which has not been changed after combined treatment with both protectants (Fig. 5a, b). We obtained similar results in the model of 6-OHDA-induced UN-SH-SY5Y cell damage (Fig. 6a). In RA-SH-SY5Y cells, we also observed that Nec-1 attenuated the cell injury evoked by 6-OHDA and that this effect was not changed by combined treatment with caspase-3 inhibitor. The latter compound was not protective in this cell phenotype (Fig. 6b).

### The Effect of Combined Treatment with Nec-1 and Curcumin in UN- and RA-SH-SY5Y Cells

In UN-SH-SY5Y cells, curcumin (5 μM) and Nec-1 (40 μM) were protective against H_2_O_2_- or 6-OHDA-evoked cell damage to the same extent when given separately or in combination (Figs. 5c and 6c). However, in RA-SH-SY5Y we observed a significantly higher protection of combined treatment with both agents when compared with the effect of each compound given alone in the model of H_2_O_2_-induced cell damage (Fig. 5d) but not in 6-OHDA one (Fig. 6d). It should be noted that Curc (5 μM) was not protective in RA-SH-SY5Y cells against H_2_O_2_- or 6-OHDA-evoked cell damage when given alone (Figs. 5d and 6d).

### The Effect of Combined Treatment with Nec-1 and Caspase Inhibitor in HT-22 Cells

**Fig. 7:**
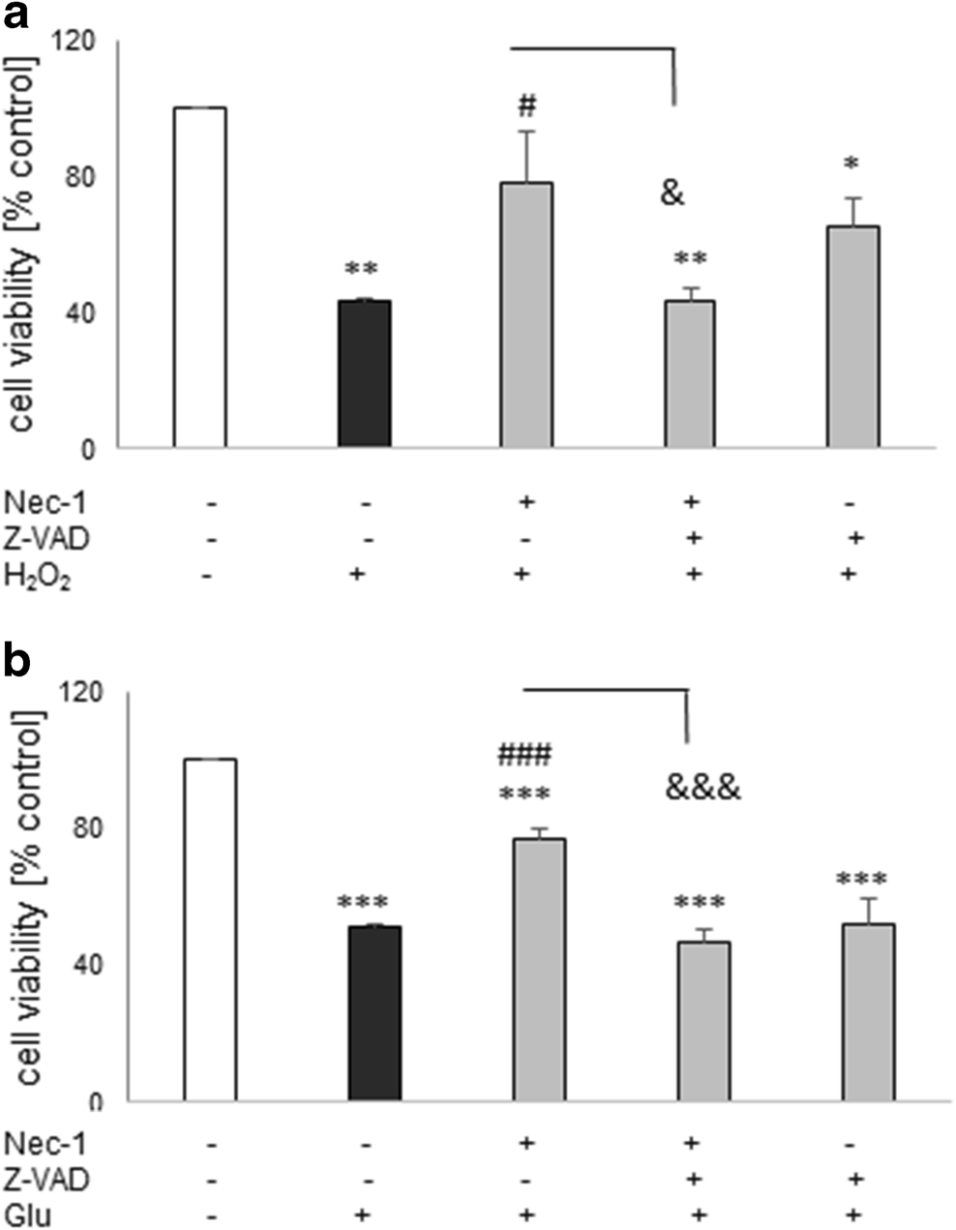
The effect of combined treatment with necrostatin-1 (Nec-1) and pan-caspase inhibitor (Z-VAD-fmk) against the H_2_O_2_- (**a**) and glutamate- (Glu, **b**) induced cell damage in HT-22 cells. The cells were pre-treated for 30 min with Nec-1 (20 μM) and Z-VAD-fmk (20 μM) alone or in combination followed by 24 h of treatment with H_2_O_2_ (1 mM) or Glu (3 mM). Cell viability was estimated by the MTT reduction assay, and data were normalized to vehicle-treated cells (control) and are presented as the mean ± SEM from 3 to 7 separate experiments with 5 repetitions each. *P < 0.05, **P < 0.01, and ***P < 0.001 vs. vehicle-treated cells; ^#^*P* < 0.05 and ^###^*P* < 0.001 vs. H_2_O_2_- or Glu-treated cells; ^&^*P* < 0.05 and ^&&&^*P* < 0.001 vs. H_2_O_2_ + Nec-1 or Glu + Nec-1-treated cells

The H_2_O_2_-evoked HT-22 cell damage was attenuated by Nec-1 (20 μM) but not by the caspase inhibitor, Z-VAD-fmk (20 μM) (Fig. 7a). Unexpectedly, the latter agent inhibited the Nec-1-mediated protection (Fig. 7a). Similar results we found in the model of Glu-induced cell damage (Fig. 7b).

### Neuroprotection Mediated by Nec-1 in SH-SY5Y Cells Is Not Connected with Caspase-3 Inhibition

**Fig. 8:**
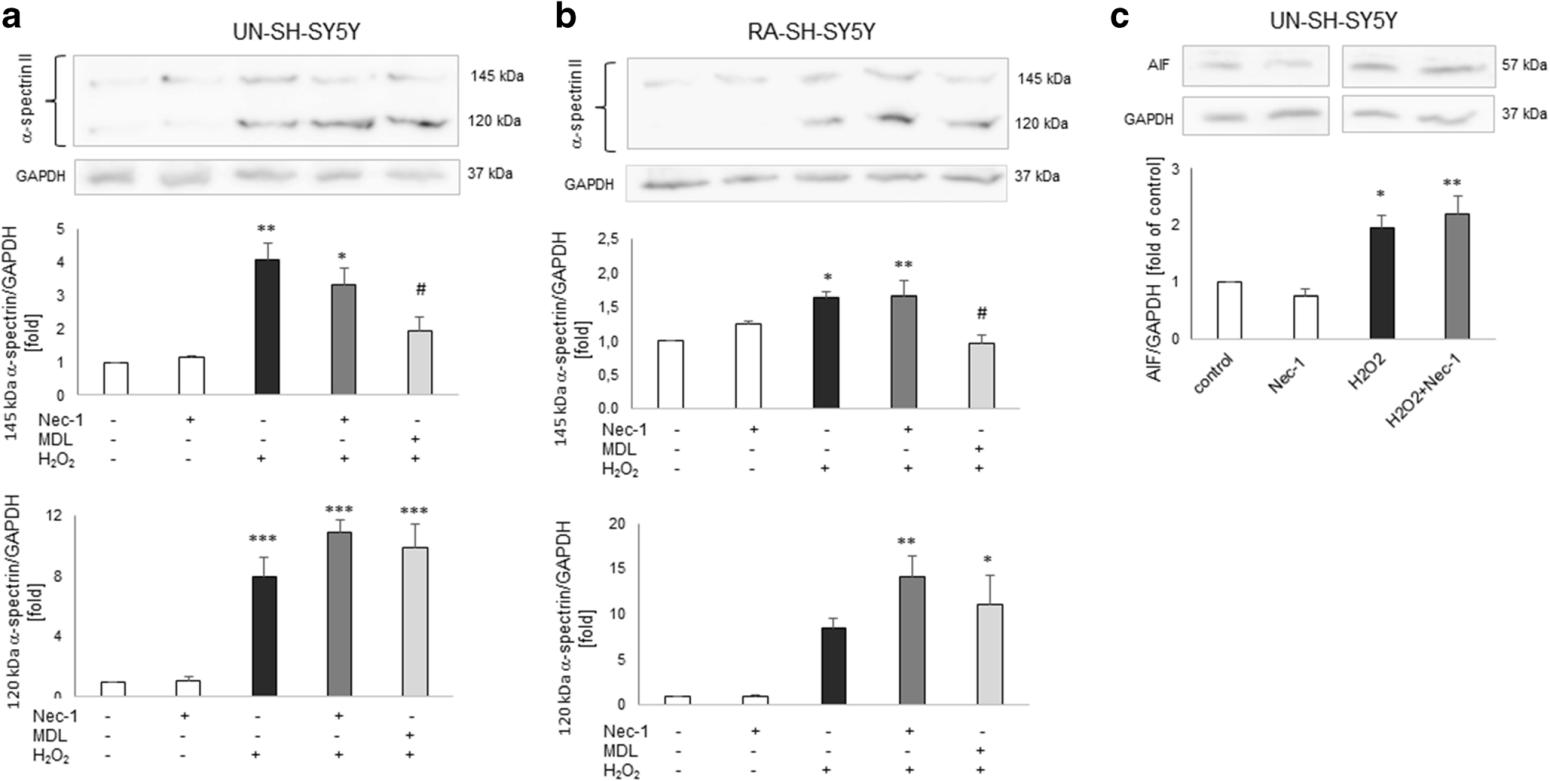
**a**, **b** The effect of necrostatin-1 (Nec-1) on the H_2_O_2_-induced 145 kDa and 120 kDa spectrin α II breakdown products in UN- and RA-SH-SY5Y cells, which are specifically cleaved by calpains and caspases, respectively. Cells were pre-treated for 30 min with Nec-1 (20 μM) or calpain inhibitor MDL28170 (10 μM), followed by 14 h of treatment with H_2_O_2_ (0.25 and 0.5 mM for UN- and RA-SH-SY5Y cells, respectively). **c** The effect of Nec-1 on the H_2_O_2_-induced increase in cytosolic AIF (apoptosis inducing factor) level. The UN-SH-SY5Y cells were pre-treated for 30 min with Nec-1 (20 μM) followed by 14 h of treatment with H_2_O_2_ (0.25 mM). **a**–**c** Histograms show the quantified Western blot results from duplicate determinations in 2–3 independent experiments which were normalized to the protein loading control (GAPDH) and are expressed as fold of the control ± SEM. *P < 0.05, **P < 0.01, and ***P < 0.001 vs. vehicle-treated cells; ^#^*P* < 0.05 vs. H_2_O_2_-treated cells

Since our previous studies showed an involvement of caspase-3 activation in detrimental effects of H_2_O_2_ in SH-SY5Y cells (Chwastek et al. 2017; Jantas et al. 2015a), and on the other hand there are data showing the effect of Nec-1 on caspase-3 activity in some models of neuronal cell injury (Wang et al. 2014; Yang et al. 2017a, b), we decided to check the effect of Nec-1 on caspase-3 activity in our study. We showed an activation of caspase-3 in the model of H_2_O_2_-evoked cell damage in UN- and RA-SH-SY5Y cells which has been totally prevented by caspase-3 inhibitor Ac-DEVD-CHO but was not affected by Nec-1 (1–20 μM) (Table 2). We further confirmed a lack of involvement of caspase-3 inhibition in neuroprotection mediated by Nec-1 against H_2_O_2_ by Western blot analysis of 120 kDa spectrin α II breakdown product which is specifically cleaved by caspases (Fig. 8a, b).

**Table 2:** The effect of necrostatin-1 on hydrogen peroxide-induced caspase-3 activity in UN- and RA-SH-SY5Y cells

	UN-SH-SY5Y	RA-SH-SY5Y	RA-SH-SY5Y
	9 h	9 h	18 h
Control	100.0 ± 8.0	100.0 ± 2.7	100.0 ± 5.8
Nec-1 20	95.9 ± 0.3	109.3 ± 5.2	119.8 ± 5.9
H_2_O_2_	753.8 ± 20.5 ***	229.6 ± 18.9 ***	487.9 ± 44.9 ***
+ Nec-1 1	854.7 ± 31.5 ***	236.9 ± 5.8 **	668.0 ± 64.3 ***
+ Nec-1 10	746.1 ± 39.2 ***	252.9 ± 12.2 ***	568.1 ± 52.8 ***
+ Nec-1 20	760.9 ± 19.8 ***	271.9 ± 8.2 ***	520.1 ± 27.5 ***
+ Ac-DEVD-CHO	33.5 ± 1.1 ^###^	24.0 ± 14.0 ^###^	19.9 ± 8.9 ^###^

UN- and RA-SH-SY5Y cells were pre-treated for 30 min with necrostatin-1 (Nec-1; 1–20 μM) followed by 9 or 18 h of treatment with H_2_O_2_ (0.25 and 0.5 mM for UN- and RA-SH-SY5Y, respectively)

As a positive control for the assay we used Ac-DEVD-CHO (20 μM), an inhibitor of caspase-3 which was given 30 min before the cell damaging factor

Data were normalized to vehicle-treated cells (control) and are presented as the mean ± SEM from 3 separate experiments with 2 repetitions each

**P<0.01 and ***P < 0.001 vs. vehicle-treated cells

^###^*P* < 0.001 vs. H_2_O_2_-treated cells

Looking for possible mechanisms responsible for synergistic effect of Nec-1 and curcumin against the H_2_O_2_-induced cell damage, we checked caspase-3 and caspase-1 activities in RA-SH-SY5Y cells. The H_2_O_2_-induced caspase-3 activity after 18 h of treatment was not attenuated by Curc (5 μM) alone or in combination with Nec-1 (20 μM) (data not shown). However, we found an increase in caspase-1 activity after 18 but not after 9 h of treatment of RA-SH-SY5Y cells with H_2_O_2_ (0.5 mM) which was significantly attenuated by combined treatment with Nec-1 and Curc, but not when the agents were given separately (Table 3).

**Table 3:** The effect of necrostatin-1 and curcumin alone and in combination against the hydrogen peroxide-induced caspase-1 activity in RA-SH-SY5Y cells

	9 h	18 h
	(% control)	(% control)
Control	100.0 ± 0.8	100.0 ± 6.4
H_2_O_2_	93.7 ± 8.3	171.5 ± 15.8 ***
+ Nec-1	107.6 ± 3.7	143.6 ± 9.5 *
+ Curc	114.8 ± 8.1	135.1 ± 3.9
+ Nec-1 + Curc	116.3 ± 2.6	128.8 ± 5.5 ^#^
+ Inh cas-1	73.9 ± 6.8	42.6 ± 4.3 **^,###^

RA-SH-SY5Y cells were pre-treated for 30 min with necrostatin-1 (Nec-1; 20 μM) followed by 24 h of treatment with curcumin (Curc, 5 μM) and H_2_O_2_ (0.5 mM)

As a positive control for the assay we used Ac-YVAD-CHO (20 μM), an inhibitor of caspase-1 which was given 30 min before the cell damaging factor

Data were normalized to the vehicle-treated cells and are presented as the mean ± SEM from 2 separate experiments with 2 repetitions each

*P < 0.05, ***P < 0.01, and ***P < 0.001 vs. vehicle-treated cells

^#^*P* < 0.05 and ^###^*P* < 0.001 vs. H_2_O_2_-treated cells

### Neuroprotection Mediated by Nec-1 Is Not Connected with Inhibition of Calpains or Caspase-3-Independent Mechanism Engaging AIF Translocation

Since calpains, calcium-dependent intracellular proteases, have been reported to participate in the mechanisms of H_2_O_2_-evoked neuronal cell damage (Chwastek et al. 2017; Jantas et al. 2015a), we measured by Western blot the protein level of 145 kDa spectrin α II breakdown product, which is specifically cleaved by calpains. Our data showed a significant increase in calpain activity after 14 h treatment with H_2_O_2_ in UN- and RA-SH-SY5Y, which has been attenuated by calpain inhibitor MDL28170 (10 μM) but not influenced by Nec-1 (20 μM) (Fig. 8a, b).

Based on our previous findings where we showed an engagement of caspase-3 independent mechanism engaging AIF translocation in the model of H_2_O_2_-evoked cell damage in UN-SH-SY5Y cells (Jantas et al. 2015a), and on the other hand, there are data showing a connection between AIF translocation and necroptosis induction (Bollino et al. 2015; Ji et al. 2011; Xu et al. 2010b, 2016), we decided to study the effect of Nec-1 (20 μM) on cytosolic AIF protein level. We observed almost twofold increase in cytosolic AIF level after 14 h of treatment of UN-SH-SY5Y cells with H_2_O_2_ (0.25 mM) which confirms our previous data (Jantas et al. 2015a); however, this effect was not changed by Nec-1 (20 μM) (Fig. 8c).

### Neuroprotection Mediated by Nec-1 Against H_2_O_2_-Evoked Cell Damage in UN- and RA-SH-SY5Y Cells Is Connected with Cathepsin D Inhibition

**Fig. 9:**
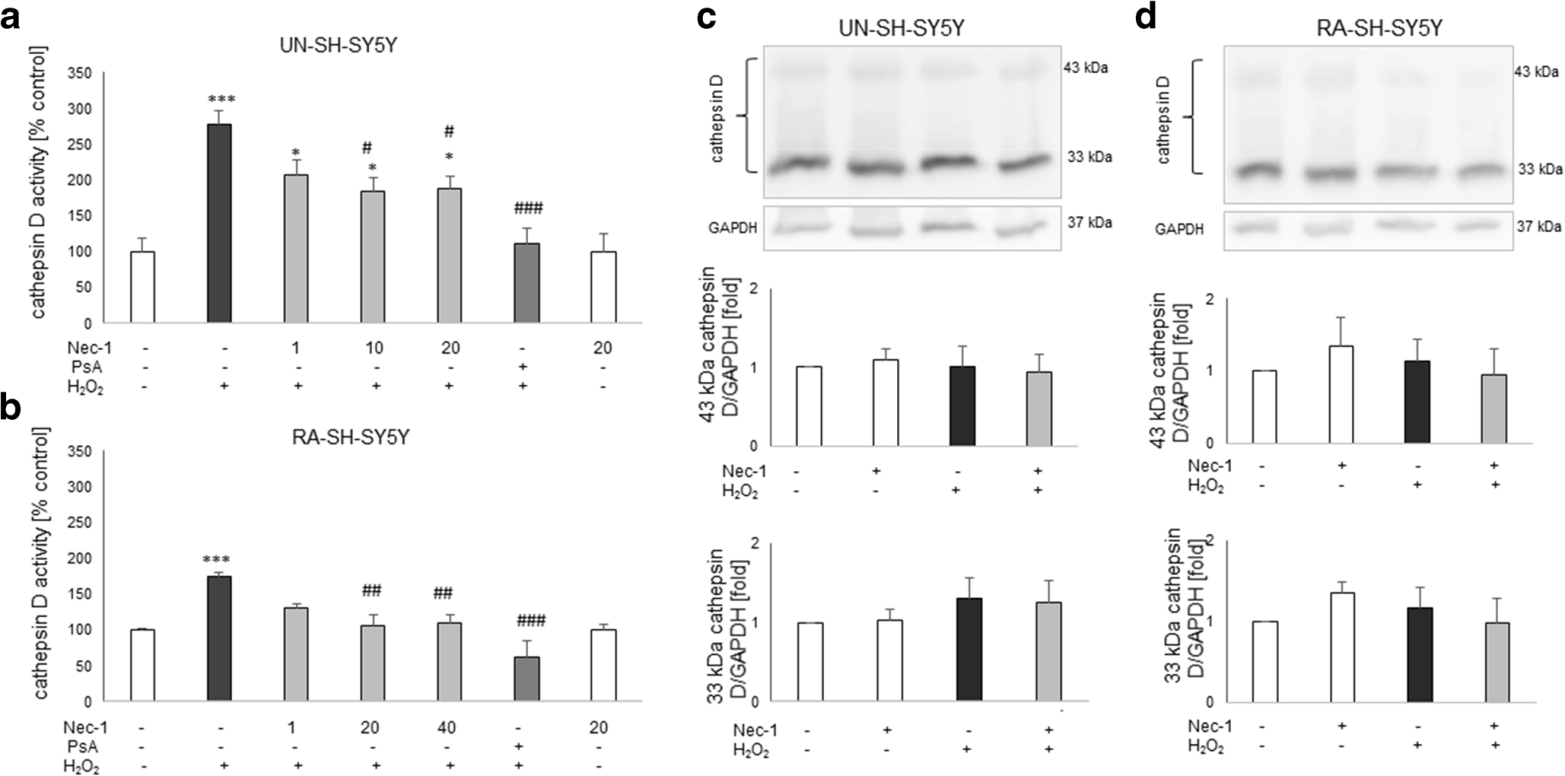
**a**, **b** The effect of necrostatin-1 (Nec-1) on H_2_O_2_-induced cathepsin D activity in UN- (**a**) and RA (**b**) SH-SY5Y cells. The cells were pre-treated for 30 min with Nec-1 (1–40 μM) or pepstatin A (PsA; 0.2 μM) followed by 18 h of treatment with H_2_O_2_ (0.25 and 0.5 mM for UN- and RA-SH-SY5Y cells, respectively). Data from duplicate determinations in 3–4 independent experiments were normalized to the protein level and are expressed as percentages of the control ± SEM. **c**, **d** The effect of necrostatin-1 (Nec-1) on H_2_O_2_-induced cathepsin D expression in UN- (**c**) and RA- (**d**) SH-SY5Y cells. The cells were pre-treated for 30 min with Nec-1 (20 μM) followed by 18 h of treatment with H_2_O_2_ (0.25 and 0.5 mM for UN- and RA-SH-SY5Y cells, respectively). Expression of 43 and 33 kDa forms of cathepsin D was done by Western blot method. Histograms show the quantified WB results from duplicate determinations in 2 independent experiments which were normalized to the protein loading control (GAPDH) and are expressed as fold of the control ± SEM

Based on our previous findings where we showed an involvement of cathepsin D activation in the cell damaging effect of H_2_O_2_ in RA-SH-SY5Y cells (Chwastek et al. 2017), we decided to check if the inhibition of this enzyme could be involved in the neuroprotective effect of Nec-1. We observed an almost three- and twofold increase in cathepsin D activity after 18 h of treatment with H_2_O_2_ (0.25 and 0.5 mM for UN- and RA-SH-SY5Y cells, respectively) which was completely blocked by cathepsin D inhibitor, pepstatin A (0.2 μM) (Fig. 9a, b). Nec-1 significantly attenuated the H_2_O_2_-evoked cathepsin D activity at concentrations of 10–20 μM and 20–40 μM for UN- and RA-SHSY5Y cells, respectively (Fig. 9a, b). We also checked the cathepsin D protein level by WB method; however, we did not find any significant changes in 43 and 33 kDa cathepsin D forms after 18 h of treatment with H_2_O_2_ and Nec-1 in UN- and RA-SH-SY5Y cells (Fig. 9c, d).

Next, we compared the level of protection mediated by Nec-1 (20 μM) or PsA (0.2 μM) when given separately or in combination in the model of H_2_O_2_-evoked cell death in UN-SH-SY5Y cells. The protection mediated by Nec-1 was comparable with the effect of PsA which was not changed after combined treatment with both inhibitors as estimated by the MTT reduction and LDH release assays (Table 4).

**Table 4:** The effect of necrostatin-1 and pepstatin A alone and in combination against the hydrogen peroxide-induced cell damage in UN-SH-SY5Y cells

	Cell viability	LDH release
	(% control)	(% control)
Control	100.0 ± 9.9	100.2 ± 6.4
H_2_O_2_	60.6 ± 4.3 ***	358.3 ± 17.2 ***
+ Nec-1	90.3 ± 4.1 ^##^	277.2 ± 14.1 ***^, ##^
+ PsA	88.9 ± 3.7 ^#^	271.8 ± 10.2 ***^, ###^
+PsA + Nec-1	89.1 ± 3.3 ^#^	280.3 ± 16.2 ***^, ##^

UN-SH-SY5Y cells were pre-treated for 30 min with necrostatin-1 (Nec-1; 20 μM) and pepstatin A (PsA; 0.2 μM) alone and in combination followed by 24 h of treatment with H_2_O_2_ (0.25 mM)

The MTT reduction and LDH release assays were employed for cell viability and toxicity assessments, respectively

Data were normalized to the vehicle-treated cells and are presented as the mean ± SEM from 3 separate experiments with 5 repetitions each

***P < 0.001 vs. vehicle-treated cells

^#^*P* < 0.05, ^##^*P* < 0.01, and ^###^*P* < 0.001 vs. H_2_O_2_-treated cells

## Discussion

The present study confirmed that SH-SY5Y cell culture is a reliable in vitro model for studying the mechanism of neurotoxicity, and that undifferentiated SH-SY5Y cells are more prone to the effects of cell damaging compounds than the differentiated ones (Chwastek et al. 2017; Jantas et al. 2014b, 2015b, 2018b; Wenker et al. 2010). We found that 30 min pre-treatment of the SH-SY5Y cells with Nec-1 in a wide range of micromolar concentrations attenuated but not totally prevented toxic effects of H_2_O_2_ or 6-OHDA suggesting that necroptosis could be only one of several activated cell death signaling pathways following exposure of the cells to the above-mentioned toxic agents. Indeed, it was reported that caspase-3-dependent apoptosis and necrosis were also involved in the mechanism of H_2_O_2_ neurotoxicity (Chwastek et al. 2017; Cole and Perez-Polo 2002; Jantas et al. 2015a; Park et al. 2016), which was also confirmed in our present study by measurement of apoptotic and necrotic markers, caspase-3 activity, and PI staining, respectively (Fig. 1, Table 2). We also found protection mediated by Nec-1 in another model of oxidative stress (6-OHDA) in UN- and RA-SH-SY5Y cells (Table 1), which confirms previous findings from PC12 cells (Wu et al. 2015), but that effect was relatively smaller than against H_2_O_2_. It could probably be explained by the predominant role of apoptotic processes in 6-OHDA-induced cytotoxicity in SH-SY5Y cells (No et al. 2010; Park et al. 2014). Our study showed that the protective effect of Nec-1 against H_2_O_2_ was quantitatively similar to the effect of the specific caspase-3 inhibitor (Ac-DEVD-CHO) in both SH-SY5Y cell phenotypes, although no synergy after concomitant treatment with the both agents could be observed (Figs. 5a, b and 6a). Similar results were obtained in the 6-OHDA model but only in UN-SH-SY5Y cells, since in differentiated ones we did not find protection by a caspase-3 inhibitor (Fig. 6b). The above findings suggest a link between apoptotic and necroptotic machinery at least in SH-SY5Y cells exposed to oxidative stress stimulus since in another cell damage model of neuroblastoma cells (induced by stenodactylin) there was a complete prevention of cell death by combined treatment with caspase inhibitor (Z-VAD-fmk) plus catalase or Nec-1 (Polito et al. 2016). Our data from SH-SY5Y cells are in contrast to results of previous study where Nec-1 (40 μM) but not pan-caspase inhibitor (Z-VAD-fmk) attenuated cell damage induced by H_2_O_2_ in neuroblastoma SK-N-SH cells (Lee et al. 2011). Similar results we obtained in HT-22 cells where Nec-1 but not the caspase inhibitor was protective against H_2_O_2_ or Glu-induced cell damage (Fig. 4) suggesting a cell-specific mechanisms of Nec-1 protective action. Moreover, in HT-22 cells, Z-VAD-fmk inhibited protection induced by Nec-1 after concomitant treatment which confirms data obtained by Xu et al. (2007) at least for the model of Glu-evoked oxytosis. However, we found an opposite effect in the H_2_O_2_-induced cell death model, since in the paper by Xu et al. (2007) they did not show Nec-1-elicited protection against oxidative stress (H_2_O_2_ or menadione)-induced cell damage. Nec-1 was also not protective against H_2_O_2_-induced cell damage in oligodendrocyte precursors (Kim et al. 2010). It is not excluded that at a shorter time of exposure to oxidative stressors (8 h) used by Xu et al. (2007) the mechanisms of cell death of HT-22 cells are more necrotic and thus not affected by Nec-1, whereas in our model with 24 h of treatment, there was the situation of necroptosis induction which could be prevented by Nec-1. Although there are some reports showing the involvement of autophagy in the H_2_O_2_-evoked cell damage of SH-SY5Y cells (Castino et al. 2010, 2011), our unpublished data showed no autophagy induction after hydrogen peroxide treatment in UN-SH-SY5Y cells (at least when measured by WB analysis of LC3 II, p62, and Beclin-1 levels after 9, 18, or 24 h after cell treatment). The differences between our results and Castino et al. (2010, 2011) findings could be explained by different concentrations of H_2_O_2_ used (250 μM and 50–100 μM, respectively) as well as culture medium content (DMEM with high glucose with 1% FBS vs. 50% minimal essential medium and 50% F12 nutrient medium supplemented with 10% FBS, respectively). All these factors could significantly influence the type of induced cell death program after the same stimulus. Since autophagy inhibition has been linked with Nec-1 neuroprotection mediated against 6-OHDA-evoked cell damage in rat PC12 cells (Wu et al. 2015), it is not excluded that this process could be also engaged in the protection against this neurotoxin in human SH-SY5Y cells.

In our study, we showed for the first time that neuroprotection mediated by Nec-1 was higher in undifferentiated than RA-differentiated SH-SY5Y cells at least when AUC values in cell viability assay were compared. This effect was more pronounced in the model of H_2_O_2_-induced cell damage than 6-OHDA-induced injury. Moreover, the non-specific apoptosis and necroptosis inhibitor curcumin significantly augmented beneficial effect of Nec-1 against H_2_O_2_, though in RA-SH-SY5Y cells only. The above facts suggest that the interplay between necroptosis and apoptosis depends on the differentiation state of the cell culture and that some mechanisms of Nec-1 neuroprotective action could be masked by differentiation of cells with retinoic acid (e.g., activation of PI3-K/Akt and MAPK/ERK1/2 pro-survival pathways) (Cheung et al. 2009; Lopes et al. 2010; Wenker et al. 2010). The higher basal and stimulated caspase-3 activity in UN-SH-SY5Y cells, shown by us, also supports anti-apoptotic phenotype of RA-SH-SY5Y cells. It is also corroborated by our observation of the lack of neuroprotective effect of a caspase-3 inhibitor or curcumin in RA-SH-SY5Y cells against 6-OHDA-evoked cell damage, which is the cell death model connected rather with apoptotic than necrotic processes that are saturated after differentiation with RA (Cheung et al. 2009; No et al. 2010; Park et al. 2014). Our results obtained in UN-SH-SY5Y cells confirmed previous findings on neuroprotective effect of curcumin which could be mediated by its antioxidant, anti-apoptotic, and anti-inflammatory mechanisms (Mhillaj et al. 2019; Sang et al. 2018; Uğuz et al. 2016).

The main finding of this study is the first observation that Nec-1 attenuating effect on hydrogen peroxide-induced SH-SY5Y cell damage is associated with the inhibition of cathepsin D (Fig. 9) but not caspase-3 or calpain activities (Table 2, Fig. 8a, b). Cathepsin D is a lysosomal protease which plays a pivotal role in protein catabolism, having an impact on several pathophysiological processes, such as organ development, neurodegeneration, or cancer (Follo et al. 2007). Elevated ROS level could increase permeability of lysosomes and thus can promote release of cathepsins into cytoplasm leading to activation of various cell death programs (e.g., apoptosis) (Castino et al. 2007; Follo et al. 2007). Human cathepsin D is synthesized as a precursor of approximately 53 kDa that is processed by proteolysis first into an intermediate single-chain of 48 kDa and eventually into the mature double-chain form (Crabtree et al. 2014; Follo et al. 2007). It should be noted that in our study, the inhibitory action of Nec-1 on cathepsin D was present in UN- and RA-SH-SY5Y cells and the basal activity of cathepsin D as well as expression of its double-chain form (43 and 33 kDa) did not differ between both cell phenotypes (Fig. 9). Moreover, the range of protection mediated by the cathepsin D inhibitor, pepstatin A, was similar to the effect of Nec-1 (Table 4) suggesting an interplay between necroptosis and lysosomal-permeability-induced cell death and a shared mechanism of action for both inhibitors. To our knowledge, there was only one report showing that necroptotic pathway was augmented by the activation of cathepsin D; however, that paper concerned activation of innate antiviral immune response in A549 cells (Wang et al. 2016). However, there are some reports linking Nec-1-mediated protection with its inhibitory effect on cathepsin B activity as has been shown in hippocampal neuronal programmed necrosis induced by ischemia/reperfusion injury (Yin et al. 2015) or in PC12 cells exposed to 6-OHDA (Wu et al. 2015). The activation of various forms of cathepsins seems to be specific for cell damaging factor and/or cell type since the activation of cathepsin D but not cathepsin B was observed in PC12 cells exposed to H_2_O_2_ (Lee et al. 2007) which was also confirmed in our previous study with SH-SY5Y cells (Chwastek et al. 2017). The active role of cathepsin D in oxidative stress–induced cell death in neuroblastoma cells was shown by siRNA-mediated downregulation of the enzyme, besides using PsA (Castino et al. 2008, 2010, 2011). One could doubt about cell permeability to PsA and efficient cathepsin D inhibition in lysosomes (Nicotra et al. 2010); however, this inhibitor has been used for many years by various research groups mostly in cell-based system (Chahory et al., 2010; Crabtree et al. 2014; Follo et al. 2007; Kanamori et al. 1998). Since cathepsin D has also physiological functions, maybe for neuroprotection strategies it will be more relevant to inhibit activity of this enzyme only when it is pathologically released from lysosomes. One could ask what mechanisms are responsible for induction of cathepsin D activity by H_2_O_2_ and inhibitory effect of Nec-1 on this parameter. By WB analysis of two mature cathepsin D forms (43 and 33 kDa), we excluded the possibility of the impact of H_2_O_2_ and Nec-1 on expression of this protein (Fig. 9c, d). It is likely that Nec-1 could chemically interact with the active site of cathepsin D in a similar way as PsA do; however, we did not find relevant reports. The only study regarding Nec-1 specificity tested its effect on the panel of 98 kinases and found that Nec-1 < 30 μM inhibited only RIP1 (Biton and Ashkenazi 2011). It will be also interesting to study in the future whether curcumin engages cathepsin D inhibition in its protective action against oxidative stress.

Among other possible mechanisms which could be involved in Nec-1-mediated neuroprotection in SH-SY5Y cells, we excluded inhibition of caspase-3 (Table 2), calpains (Fig. 8a, b), or AIF translocation (Fig. 8c) although all of them were induced by H_2_O_2_. Some reported data pointed to a connection between AIF translocation and necroptosis induction (Bollino et al., 2015; Ji et al. 2011; Xu et al. 2010b, 2016) and that Nec-1 inhibited the nuclear translocation of AIF in the model of Glu-evoked cell damage (Xu et al. 2007). Previously, we described a caspase-3-independent mechanism engaging AIF translocation in the model of H_2_O_2_-evoked cell damage in UN-SH-SY5Y cells (Jantas et al. 2015a). Although the present data confirmed AIF translocation in the oxidative stress model, it was not affected by Nec-1 treatment. It should be underlined that we observed the impact of curcumin on H_2_O_2_-induced caspase-3 activation in UN-SH-SY5Y cells (data not shown) which could be a result of its ROS-scavenging activity (Szczepanowicz et al. 2016) and consequently inhibition of apoptotic processes. It should be mentioned that once translocated into the cytosol upon oxidative stress–induced permeabilization of the lysosomes (H_2_O_2_ for 30 min), cathepsin D was shown to act on Bax to induce apoptosis in human neuroblastoma cells (Castino et al. 2007). However, this phenomenon is unlikely to be implicated in Nec-1 neuroprotective action against H_2_O_2_-evoked cell damage since under our experimental conditions cathepsin D was activated relatively late (after 18 h, Chwastek et al. 2017) whereas apoptotic changes occurred earlier (9 and 18 h) and were not changed by Nec-1 (Fig. 3b, c, Table 2). It is interesting that we found the influence of Nec-1 on H_2_O_2_-evoked neurite shortening (Fig. 3d) which confirms in vivo findings on participation of necroptosis in axonal degeneration and suggests a possible therapeutic intervention by its inhibition by Nec-1 (Arrázola et al. 2019). While this effect was observed in UN-SH-SY5Y at the late stage (18 h), in RA-SH-SY5Y cells, it was present at both studied time points (9 and 18 h) (Fig. 3d). The mechanisms by which Nec-1 interfere with H_2_O_2_-evoked reduction of neurites length are unknown yet and should be investigated in the future. It should be underlined that RA-differentiated cells are more suitable for monitoring of neurite outgrowth than undifferentiated ones (Lee et al. 2015; Miloso et al. 2004). Finally, we confirmed in RA-SH-SY5Y cells the H_2_O_2_ potency to induce ICE-like protease, caspase-1 activity (Jantas et al. 2018a) which was significantly attenuated only by Nec-1 + Curc combination (Table 3). Activation of this enzyme participates in another form of cell damage, namely pyroptosis (Espinosa-Oliva et al. 2019; Wang et al. 2019). Since previous data showed the activation of this pro-inflammatory protease in neuronal cell type, including SH-SY5Y cells and neuroprotective potency of its inhibitors (Chen et al. 2019; Knaryan et al. 2014; Nath et al. 1996; Zhao et al. 2019a, b), it is highly probable that this mechanisms could be responsible for synergistic protective action of Nec-1 and curcumin against H_2_O_2_-evoked cell damage, observed in the present study.

**Fig. 10:**
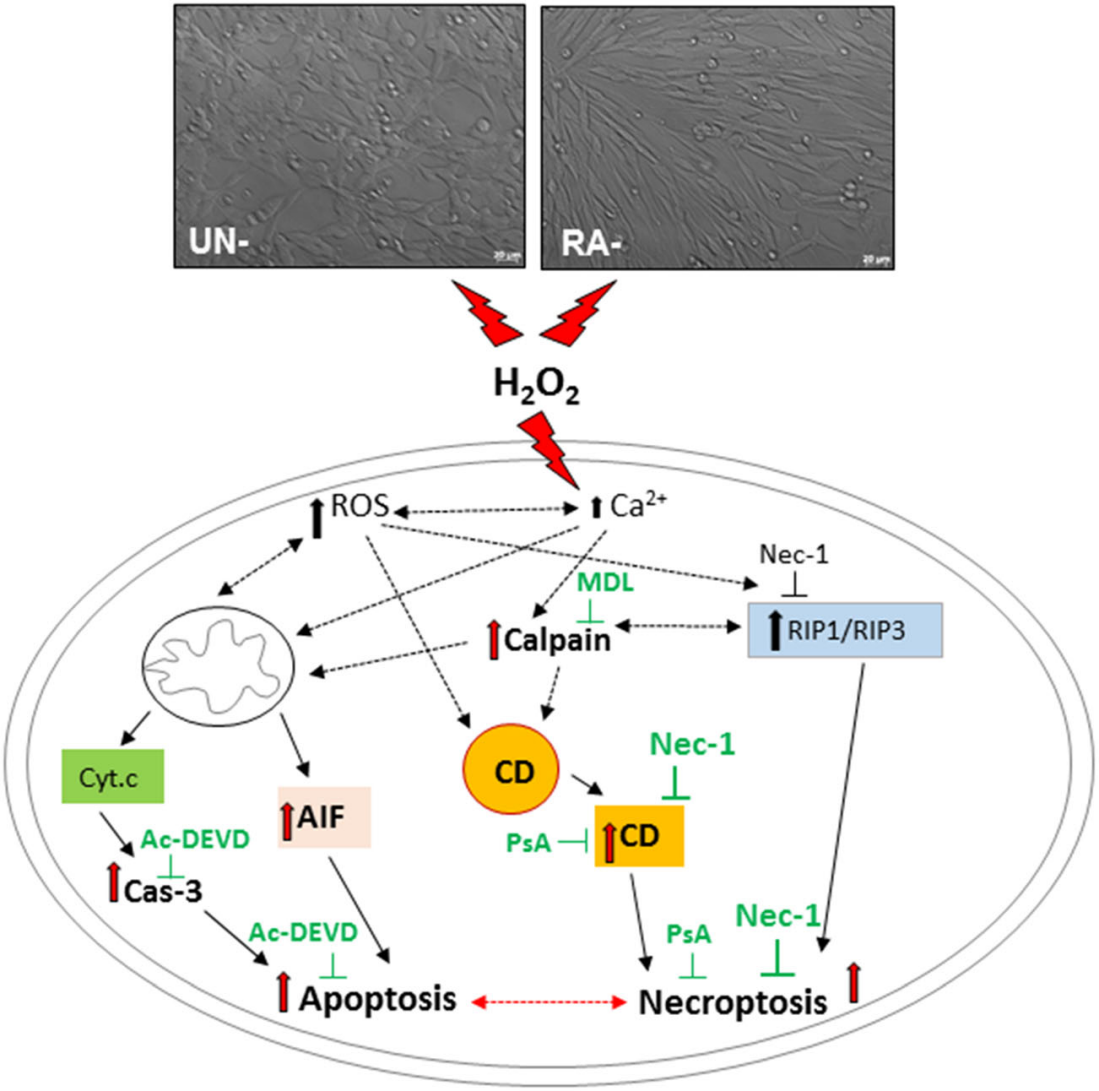
A schematic illustration of possible mechanisms by which the necrostatin-1 (Nec-1) mediates neuroprotection against the H_2_O_2_-induced cell damage in undifferentiated (UN-) and retinoic acid (RA)-differentiated SHSY5Y cells. Cathepsin D inhibition, but not caspase-3, calpain or AIF (apoptosis inducing factor) translocation inhibition, is proposed as a candidate mechanism associated with neuroprotection mediated by Nec-1 in oxidative stress (H_2_O_2_)-evoked cell damage. Ac-DEVD—Ac-DEVD-CHO, an inhibitor of caspase-3; MDL—MDL28170, an inhibitor of calpains; CD—cathepsin D; PsA—pepstatin A, an inhibitor of cathepsin D; H_2_O_2_—hydrogen peroxide; ROS—reactive oxygen species

Collectively, our data showed neuroprotective effects of the necroptosis inhibitor, Nec-1 against oxidative stress–induced cell damage, and pointed to involvement of cathepsin D inhibition in the mechanism of its action. Moreover, a cell type–specific interplay between necroptosis and apoptosis has been demonstrated (Fig. 10).
